# Barriers and facilitators of sport and physical activity for Aboriginal and Torres Strait Islander children and adolescents: a mixed studies systematic review

**DOI:** 10.1186/s12889-020-8355-z

**Published:** 2020-05-01

**Authors:** Tamara May, Amanda Dudley, James Charles, Kate Kennedy, Ana Mantilla, Jane McGillivray, Keane Wheeler, Hope Elston, Nicole J. Rinehart

**Affiliations:** 1 Paediatrics Monash Health, Monash University, Clayton, Victoria Australia; 2 School of Psychology, Deakin University, Burwood, Victoria Australia; 3 Institute of Koorie education and School of Medicine, Deakin University, Waurn Ponds, Victoria Australia; 4 Oodgeroo Unit, Queensland University of Technology, Brisbane, Queensland Australia

**Keywords:** Aboriginal and Torres Strait islander, Sport, Physical activity, Barrier, Facilitator

## Abstract

**Background:**

Participation in sport and physical activity could minimise the inflated risk of poor physical health outcomes for Aboriginal and Torres Strait Islander children and adolescents. This review aimed to synthesise existing quantitative and qualitative literature regarding barriers and facilitators to physical activity and sports participation in Aboriginal and Torres Strait Islander children.

**Methods:**

Literature was systematically searched to include studies reporting barriers or facilitators to physical activity and/or sports participation in Aboriginal and Torres Strait Islander children aged 0–18 years. Using a pre-established taxonomy based on the social-ecological model, a deductive analysis was performed. Quality appraisal was performed using the Mixed Methods Appraisal Tool.

**Results:**

Of 3440 unique articles, nine studies were included with *n* = 10,061 total participants. Of the nine included studies one reported on participants from urban areas, two from regional and three from remote areas. Three were from representative samples of the Aboriginal and Torres Strait Islander population. Barriers were reported in all nine studies: 18 individual, 9 interpersonal, 27 community and 4 at the policy level (58 total); Facilitators were reported in five studies: 12 individual, 11 interpersonal, 11 community and 3 policy level (37 total).

**Conclusions:**

Research in this area is lacking with some states in Australia not represented and small samples. Strategies for improving participation in sport and physical activity by Aboriginal and Torres Strait Islander children and adolescents need to integrate a comprehensive identification of barriers and facilitators with a social-ecological understanding of how community and cultural factors can impact individual participation.

## Background

Sport and physical activity (PA) are important to many Aboriginal and Torres Strait Islander cultures. For millennia, ball games were played by many different Aboriginal Nations for enjoyment, where the entire community would participate [1, 2]. Participation in sport and PA for many Aboriginal and Torres Strait Islander People is often paramount to belonging and taking part in cultural activities [3]. The modern culture of Aboriginal and Torres Strait Islander sport is no more evident than with the participation in various State and National, Aboriginal National Rugby League (NRL), Australian Football League (AFL), netball and cricket carnivals for men, women and children, which are large cultural events [4–6].

There is ample evidence of the benefits for children and young people that participate in the recommended amount of PA, including improved sleeping patterns, skeletal health, social, psychological and cognitive health, especially for those under 11 years of age [7–9]. Aboriginal and Torres Strait Islander children and adolescents however experience reduced physical health and wellbeing relative to non-Indigenous children [10–12]. Some research has found that Aboriginal and Torres Strait Islander children may initially engage in more PA than non-Indigenous children, but this appears to reduce with age, such that Aboriginal and Torres Strait Islander adults are less active than their non-Indigenous counterparts [13]. Aboriginal and Torres Strait Islander People at higher risk of developing preventable diseases such as type-2 diabetes [14], obesity [11, 15] and higher psychological distress and mental health problems than non-Indigenous children [16, 17].

Research that looks specifically at Aboriginal and Torres Strait Islander People’s participation in sport and PA is limited [18–21]. Some studies have shown that participation in PA like hunting and traditional dance has been shown to provide important cultural connections for Aboriginal and Torres Strait Islander People [22]. Similarly, positive associations with physical and mental health have been reported. Research by Dalton, Wilson, Evans and Cochrane [18] found that Aboriginal and Torres Strait Islander young people who participated in sport were 3.5 times more likely to report good general health and 1.6 times more likely to have no probable serious mental illness than those who did not participate in sport [23]. In a qualitative study of six Aboriginal and Torres Strait Islander adolescents undertaken by Fitch, Ma’ayah, Harms and Guilfoyle [24], the influence of sports participation in secondary school on their lives was retrospectively reported. Involvement in sport positively influenced a wide range of areas including their motivation for education, school engagement, planning and decision-making, interpersonal skills and development of a more positive and empowered identity. However, given the study involved a small number of participants from a single setting, these purported benefits need to be replicated in larger studies.

Sport and PA are being employed as vehicles for improving social and health outcomes in disadvantaged populations [18, 25]. In Australia, the ‘Sport, more than just a game’ report (2013) demonstrates that sport is being used as an important lever to ‘Close the Gap’ [26, 27]. The general evidence of improved mental health from participation in sport and PA suggests that increasing participation may help to reduce high rates of youth mortality. Improvement in mortality rates for Aboriginal youth would also see a reduction in the “life expectancy gap” for Aboriginal and Torres Strait Islander People, which has increased from 10.2 to 10.8 years for males and 9.6 to 10.6 years for females in 2019 [28]. The Close the Gap 2019 report shows suicide as the 5th leading cause of death for Aboriginal and Torres strait Islanders People and the only leading cause of death to actually increase in 2018 [29]. Mortality rates of Aboriginal adolescents are twice that of non-Indigenous adolescents, predominantly through intentional self-harm, with 80% of adolescent deaths considered to be preventable [12]. There has therefore never been a greater need to improve the social and emotional health of Aboriginal and Torres Strait Islander People and improving access to sport and PA could play an integral role to this improvement. Understanding the barriers and facilitators to participation in sport and PA for children and adolescents can guide the development and implementation of tailored programs and contribute to existing initiatives aimed at improving Aboriginal and Torres Strait Islander health and wellbeing through PA programs [30]. This is particularly important as young people transition to adulthood where they are at higher risk of inactivity, reduced quality of life and chronic disease [13].

There may be a range of individual, family and community factors which can influence participation in sport and PA in Aboriginal and Torres Strait Islander children and adolescents. Various models such as the social-ecological model have been used to explore barriers and facilitators to sport and PA [31, 32]. In general terms, social-ecological models seek to understand the interactive effect of individual characteristics, interpersonal processes, institutional factors, community features and public policy on behaviour. A prior study of Aboriginal and Torres Strait Islander People used the social-ecological model to understand PA engagement [32]. This narrative review utilised different levels of the model to examine how individual engagement in sport and PA is influenced by cultural and environmental factors and vice versa. For example, macro-social factors including colonialism, discrimination and dispossession were identified as negatively impacting on Aboriginal and Torres Strait Islander health. At the same time, at the individual level, some urban Aboriginal and Torres Strait Islander young people reported that they used PA to manage stress. Differing cultural views on PA and exercise were identified: From a Western perspective the concept of exercise was connected with an individual taking care of their health, whereas engagement in PA for Aboriginal and Torres Strait Islander People often related to social roles and communal activities [33]. Economic and social disadvantage were identified as limiting opportunities for young Aboriginal and Torres Strait Islander People to participate in sport and PA. For example, some young Aboriginal and Torres Strait Islander People needed to work to support themselves, limiting their time for recreation. However, recreation and PA were also identified as providing positive social support for young people who experienced family dysfunction. While barriers and facilitators were not systematically explored in this review it highlighted the complex interaction of social-ecological levels which affect the engagement of Aboriginal and Torres Strait Islander People in PA [32].

This systematic review aims to address the gaps in prior research through: 1) collating and synthesising the findings of research conducted to date that has explored the barriers and facilitators to participating in PA and sport for Aboriginal and Torres Strait Islander children and adolescents and 2) evaluating the quality of the studies conducted. This is the first published review to synthesise the available evidence on the barriers and facilitators of sport/PA participation for Aboriginal and Torres Strait Islander children and adolescents. Findings can therefore have the potential to inform practice and support the development of empirical studies and program evaluations undertaken in tandem with the implementation of PA programs designed with and for Aboriginal and Torres Strait Islander People [18, 21, 27, 30, 33].

## Methods

### Eligibility criteria

Original research whereby results of primary studies of any design reporting barriers and facilitators associated with participating in sport or PA for Aboriginal and Torres Strait Islander People from birth to 18 years of age. There was no limitation on the country where the research was performed, but the research participants had to be Australian Aboriginal and Torres Strait Islander children and adolescents. All published and unpublished studies, government reports, including conference proceedings and publications of full-text papers were included. Studies which did not report barriers and facilitators separately for Aboriginal and Torres Strait Islander children were excluded. Studies where adults (parents/family/community members and service providers) reported barriers and facilitators were included if these specifically related to children and adolescents’ participation in sport and PA. Qualitatively reported barriers and facilitators, as well as those reported quantitatively through proportions endorsed, were included.

### Study identification

The following databases were searched for references from 1 Jan 1946 to 31 March 2018: CINAHL Complete via Ebsco, Cochrane database, EMBASE, ERIC via Ebsco, Medline Complete via Ebsco, Prospero, PsycINFO via Ebsco, PubMed, Scopus, and SPORTDiscus Full Text via Ebsco. Bibliographies of included articles were hand-searched to identify further published, unpublished and ongoing studies. Additional health and Indigenous-specific databases were searched to identify grey literature, such as government reports, relating to the study topic, as was Google Scholar and Informit (Health and Indigenous subsets). Google (google.com.au) was also searched on 4/03/2019 to find additional grey literature not identified in the former searches. The Australian Indigenous Health Bulletin Physical Activity (http://healthbulletin.org.au/category/topics/physical-activity/) and the Australian Indigenous HealthInfoNet (healthinfonet.ecu.edu.au) were also scanned in detail on 6/3/2019 and 20/09/2019 respectively. The search focused on the following population: “Aboriginal” OR “Torres Strait Islander” OR “First People” OR “First Australian” OR “Indigenous” AND “Australia” (including all States and “Cape York” and “Arnhem Land” as keywords) AND keywords for age ranging from “infant” to “youth” OR “adolescent” AND contexts: “Sport” OR “Recreation” OR “Physical Activity” including various keywords like “movement”, “game” and a variety of sports (e.g. football, cricket, rugby, etc.). Two reviewers independently assessed all study titles and abstracts to determine inclusion, with the full text being subsequently retrieved for potentially eligible studies to assess final suitability according to inclusion criteria and by confirming that barriers and facilitators associated with participating in sport or PA for Aboriginal and Torres Strait Islander People from birth to18 years of age were being reported. Discrepancies that could not be resolved between the two reviewers were discussed with a third reviewer. The aim was to be as inclusive as possible and hence studies were retained regardless of the rigour of their research methodology.

### Data extraction

Two reviewers, working independently, used a standardised form to extract methodological, demographic, and outcome data. Data extracted included reported child/youth characteristics (number of participants, participant age range, gender), the location of studies, study aims, study methods and reported barriers and facilitators. Barriers and facilitators to PA and sport were coded based on a social-ecological model with four interactive levels:

1. Individual— identified barriers and facilitators in relation to personal attitudes, motivations and self-efficacy as well as gender, racial identity and economic status;
2. Interpersonal— identified barriers and facilitators in relation to family, friends, peers and other social support systems as well as connection with cultural practices;
3. Community—identified barriers and facilitators in relation to the provision of sporting services, facilities, equipment and transport. Climate and environment factors were also included in this category as well as connection to community leaders, sports staff and coaches;
4. Policy/ Institutional—identified barriers and facilitators in relation to government policy and programs as well as community led and implemented programs [34].

A taxonomy using this model and past research on barriers and facilitators for sports and PA (Moore 2010, Martins 2015) with codes expanded upon through reading the full articles was developed. Two reviewers independently read each publication and identified the unit of text (a sentence or paragraph representing one idea) relating to barriers or facilitators to sport/PA. The text was then coded according to the established taxonomy in a Microsoft Excel spreadsheet. Units of text which could not be coded were discussed by the two reviewers and new codes created if necessary, thus refining and expanding the preliminary list of codes. Discrepancies between the coders were resolved through iterative discussions. Codes were placed under the relevant level within the social-ecological framework. Some studies reported barriers and facilitators based on percentage of endorsement from the study participants. We included only the reported barriers and facilitators that had at least 20% endorsement by participants.

### Review protocol

This study followed the PROSPERO guidelines and a detailed protocol is available at PROSPERO (identifier CRD42018113054).

### Quality appraisal

Papers that meet the selection criteria were critically assessed for methodological quality and the extent to which a study had addressed the possibility of bias in its design, conduct and analysis using the Mixed Methods Appraisal Tool (MMAT) 2018 [35]. The MMAT is designed for the appraisal of mixed studies reviews, which include qualitative, quantitative and mixed methods studies. The MMAT has two screening questions for all study types then five questions for each of five possible types of study designs to assess the quality of studies.

## Results

### Study selection

**Fig. 1:**
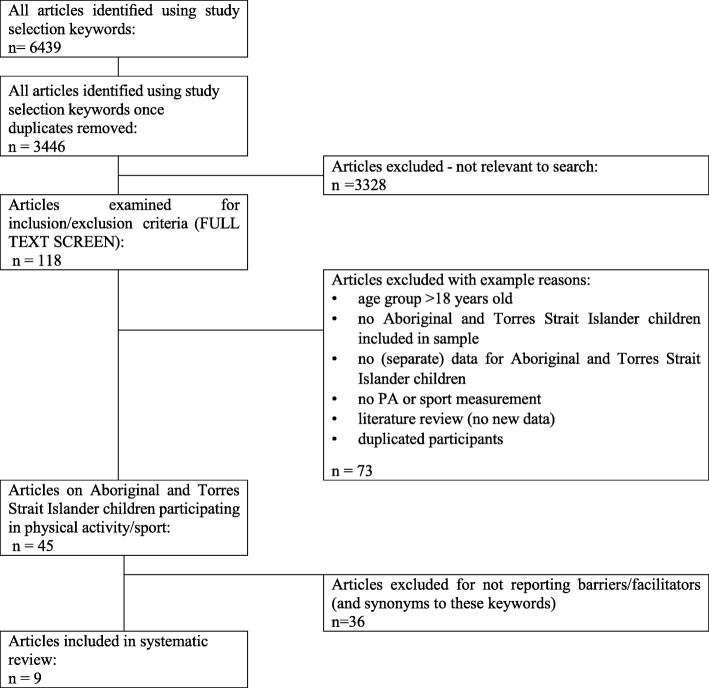
Identification, screening, eligibility and inclusion of studies

There were 6442 studies identified with 3440 after duplicates were removed. Nine studies met inclusion criteria (see Fig. 1).

There were five studies reporting barriers and facilitators derived from thematic analysis [34, 36–39] and four reporting quantitatively measured barriers and facilitators [25, 33, 40, 41]. Table 1 delineates the main characteristics of these studies.

**Table 1:** Summary of included qualitative and quantitative studies reporting barriers and facilitators associated with sport/PA participation

Authors	Year	Participant Number	Research Participants	Age of children and/or adolescents in study	Study Location	Study type	Method (e.g. individual interviews; focus groups; surveys	Analysis	Aims relating to physical activity/ sport
Abbott, Jenkins, Haswell-Elkins, Fell, MacDonald, & Cerin	2008	*n* = 367 F = 184 M = 183	Torres Strait Islander children	9–16 years	Remote - Torres Strait Islands and Northern Peninsula Area of Far North Queensland	Quantitative	Survey	Survey based on New South Wales Schools Fitness and Physical Activity survey	Understand physical activity and fitness practices
Dockery A.M. & Gorman, S.	2017	4156	Parents and guardians of Aboriginal and Torres Strait Islander children	4–14 years	Victoria, South Australia, Western Australia, Tasmania and the Northern Territory	Quantitative	National Aboriginal and Torres Strait Islander Social Survey 2014–15	Multivariate analysis	Factors that determine participation in sport; and participation in AFL only
Edwards, N. Coffin, J., & Lower, T.	2005	*n* = 15	Aboriginal adolescents	12–16 years	Geraldton, Western Australia	Qualitative	Photovoice (photos and interviews)	Qualitative analytical approach not described.	Barriers and enablers to participation in physical activity
Evans, J. R, Wilson, R., Coleman, C., Man, W.Y.N., & Olds, T.	2018	Not reported	Aboriginal and Torres Strait Islander children and adolescents	5–17 years	Remote and non-remote Australia	Quantitative	Australian Aboriginal and Torres Strait Islander Health Survey 2012–13	Secondary data analysis	Levels of physical activity in remote and non-remote areas; and PA variations associated with gender, age and SES
Gwynn	2014	Not reported	Aboriginal & non-Indigenous Community members reporting on children in school years	5–8 years	Regional - New South Wales – Taree & Kempsey	Qualitative	Focus groups with community members	Thematic analysis approach not described.	Barriers to physical activity participation
MacDonald, Abbott & Jenkins	2012	*n* = 21 Families	Torres Strait Islander families – mothers & their daughters	Not reported	Remote -Torres Strait Islands	Qualitative	Family Interviews (semi-structured & open).	Thematic analysis.	Thoughts about physical activity by girls/women
Meldrum & Thompson	2012	*n* = 4	Representatives from sporting organisations	Not reported	Remote - Cape York	Qualitative	Individual interviews	Thematic Analysis (Patton 2002)	Enablers and barriers to each sporting organisation’s operation in the communities
Nelson	2009	*n* = 14 F = 57.15% M = 42.85%	Aboriginal and Torres Strait Islander children	11–13 years	Urban - Queensland	Qualitative	Individual interviews	Thematic Analysis (Patton 2002)	Perceptions of sport and Physical activity
Victorian Department of Education and Early Childhood Development (DEECD)	2009	*N* = 5484 children Male/ female breakdown for children not reported	Aboriginal and Torres Strait Islander parent/caregiver reports on children	4–14 years	Australia	Quantitative	Survey via individual proxy interviews with parents/ guardians	2008 National Aboriginal and Torres Strait Islander Social Survey (NATSISS), Australian Bureau of Statistics	Reasons for not playing organised sport

### Participants

There were 10,061 participants in total which included 54 participants (21 counted as ‘families’) in qualitative studies (with two studies not reporting the number of participants) and 10,007 in two quantitative studies. Participants came from Western Australia (WA), New South Wales (NSW) and Queensland including the Torres Strait Islands, Far North Queensland, Cape York and the Northern Peninsula area, and two Australia wide. One study reported participants from urban areas, two from regional areas, three from remote areas and two studies reported an Australian geographically representative sample. Five studies included self-reports of children on barriers and facilitators: one study included self-reported barriers and facilitators for 21 family groups [38]; one study reported barriers and facilitators identified by Aboriginal and Torres Strait Islander youth in their community [41]; one reported on barriers as noted by community members [37]; one reported barriers and facilitators of organisations providing sporting programs for children [39]; and one investigated barriers reported by parents/guardians for their child [33]. Studies were published between 2005 and 2018. Six studies included both males and females, one study included females only and two studies did not report the sex of participants.

### Quality appraisal

Table 2 shows the results from the quality appraisal. Six of the nine studies had no more than one negative rating, and one had five of six negative ratings indicating low quality. This was primarily due to a lack of details regarding the study methodology.

**Table 2:** MMAT quality appraisal results ^a^

	1. Qualitative					4. Quantitative descriptive				
	1.1	1.2	1.3	1.4	1.5	4.1	4.2	4.3	4.4	4.5
	Approach appropriate to the research question	Data collection methods adequate	Findings adequately derived from data	Interpretation of results substantiated by data	Coherence between the data source, collection, analysis, interpretation	Sampling strategy relevant to the research question	The sample representative of the population	Measurements appropriate	Risk of nonresponse bias low	Statistical analysis appropriate to the research question
Abbott, R. et al. (2008) [33]						✓	✓	✓	x	✓
Dockery A.M. & Gorman, S. (2017) [41]										
Edwards, N. et al. (2005) [39]	✓	✓	✓	U	U					
Evans, J. R, et al. (2018) [25]						✓	✓	✓	U	✓
Gwynn, J. et al. (2014) [34]	✓	U	U	U	U					
MacDonald, D. et al. (2012) [36]	✓	✓	✓	✓	U					
Meldrum et al. 2012 [37]	✓	x	✓	✓	U					
Nelson, A. (2009) [38]	✓	✓	✓	✓	✓					
VIC DEECD (2009 [40])						✓	✓	✓	✓	✓

✓ Yes

x No

*U* Unclear/Unsur

^a^The Mixed Methods Appraisal Tool questions have been presented in the form of statements in Table 2

### Barriers and facilitators

Table 3 categorises barriers and facilitators using a social-ecological model. Facilitators were comparatively under-researched (seven of nine studies compared with all nine studies assessing barriers). There were 18 individual, 9 interpersonal, 27 community and 4 policy level (58 in total) barriers and 12 individual, 11 interpersonal, 11 community and 3 policy level (37 in total) facilitators. Barriers and facilitators in remote locations were noted separately within Table 3.

**Table 3:** Barriers (B) and facilitators (F) to physical activity and sport in a socioecological framework and each study contribution including remote only barriers and facilitators

Level	Description of Barrier / Facilitator	Study Reference Number									Total Number of	
		2	3	4	5	8	9	1 r	6 r	7 r	Barriers	Facilitators
Individual											18	12
	Motivation					F	B	B			2	1
	Enjoyment of Activity		F									1
	Shyness /Embarrassment		B			B		B	B		4	
	Perceived lack of ability				B			B			2	
	Lack of sporting ability					B					1	
	Financial cost/opportunity		B		B	F					2	1
	Lack of time						B	B			2	
	Goals/Future opportunities					F			F			2
	Having good health					F			F			2
	Cultural practices					F						1
	Program delivered in local language									F		1
	Preference for certain sports					BF					1	1
	Racial/ethnic identity					BF					1	1
	Alternative to negative behaviours					F						1
	Academic ability					B					1	
	Use of drugs, alcohol and smoking		B								1	
	Overweight or obese			B							1	
Interpersonal								r	r	r	9	11
	Parents who smoke	B									1	
	Living in a sole parent household	B									1	
	Moving house in the past 5 years	B									1	
	Family owns their own home	F										1
	PA important to the family							F				1
	Parental/Family role-modelling/activity level				B	BF					2	1
	Friends activity level					F		B			1	1
	Connection with friends		F									1
	Family connectedness		F			F						2
	Family provides transport					F						1
	Lack of family car	B									1	
	Peer competition					BF					1	1
	Connection with cultural practices	F				F				B	1	2
Community								r	r	r	27	11
	Living in a remote community			F								1
	Lack of sporting facilities and amenities		B		B					B	3	
	Lack of sporting equipment						B	B			2	
	Lack of readily accessible venues and equipment outside of organised sporting sessions		B								2	
	Lack of appropriate training						B				1	
	Lack of activities/programs							B	B		2	
	Type of sporting program					F			F			2
	Opportunity to hire equipment		F									1
	The distance of sporting venues from residential areas		B		B						2	
	School holiday programs with transport to physical activities		F									1
	Safety of community play areas for children				B						1	
	Poor community cohesion				B						1	
	Lack of transport		B		B					B	3	
	Experiences of racism in the community	B			B				B		3	
	Climate too hot							B			1	
	Seasonal restrictions									B	1	
	Access to natural environment, tracks and parks		F									1
	Fear of dogs							B			1	
	Community members/leaders					BF				B	2	1
	Perception of negative attitude of authorities		B								1	
	Admired sports people					F						1
	Connection to the community/sense of belonging					F						1
	Organisations providing services									F		1
	Cost of organisation providing services									B	1	
	Coaching programs poorly attended/too academic									B	1	
	Consistent mentoring of sports staff required									F		1
Policy / Institutional								r	r	r	4	3
	Welfare dependency				B				B	B	3	
	The Aboriginal community governed collaboration				F							1
	Continuity of provision of sporting/PA programs								B		1	
	Effective publicity about sporting programs by clubs and agencies		F									1
	Using a MoU to make partnership explicit									F		1

*B* barrier, *F* facilitator, *r* remote location

1 = Abbott 2008, 2 = Dockery 2017, 3 = Edwards 2005, 4 = Evans 2018, 5 = Gwynn 2014; 6 = Macdonald 2012; 7 = Meldrum 2012; 8 = Nelson 2009; 9 = Victorian DEECD 2009; r = Remote only location

Individual: At the individual level, a perceived lack of sporting ability [33] was considered a barrier. The cost of participating in organised sport [34] and the cost of public transport [34] were also barriers. Lack of motivation, time and a lack of interest to do any/more sport were also barriers [33, 40]. Being overweight or obese and seeing oneself as lacking sporting ability were identified as barriers [24, 33]. Shyness, related to wearing sports clothing or bathers (for swimming), was identified as a barrier particularly for adolescent girls [33, 39]. In one study focused on women and girls living in remote rural communities in Torres Strait and Northern Peninsula Area, participants reported a sense of ‘shame’ related to being seen to be active in public. This acted as a barrier towards their engagement in PA [36]. In this study, behaving in ways appropriate to perceived gender roles was also identified as facilitating the PA of boys and men who had the ‘freedom’ to exercise, whereas women felt they needed to prioritise their caring roles and family responsibilities [36]. Older children in more remote areas also reported not having activities organised for them as a barrier [33]. Preferences for specific sports such as rugby for boys was a barrier to playing other sports [38].

The potential for a financial reward through playing sport was seen as a facilitator [38]. Providing sport options that were viewed as appropriate for females such as volleyball was also a facilitator [36, 38]. Similarly, dance, including traditional dance was identified as a popular activity that facilitated the PA of girls and young women. Having a ‘natural affinity’ for sport, a sense of pride and collective identity were reported as facilitators [38]. Sports was seen as a ‘way out’ or as providing an opportunity for the future [36, 38]. It was also viewed as a means to prevent poor health outcomes like diabetes [36], and to steer young people away from ‘undesirable behaviour’ such as underage drinking and poor dietary choices [36, 38]. Having the program delivered in the local language was a facilitator in a remote area [37].

Interpersonal: A lack of family involvement [38] and low levels of activity in friends were barriers [33] at the interpersonal level. The transience of those conducting the programs, including teachers was also reported as a barrier [37]. The family viewing sport as essential and role modelling participation were clear facilitators [33]. Parents providing transport and financial support were also facilitators [38]. A high level of activity in friends was also a facilitator [38]. Having an affinity with certain sports or seeing PA such as dance as an opportunity to connect with cultural practices was a facilitator [38]. The competitive nature of sport acted as both as a barrier and facilitator in that some young people felt the drive to win as a motivation while others (in the same study) did not like competition because losing a game prompted feelings of shame [38].

For studies conducted in remote locations barriers included friends having a low activity level [33] and the transient nature of teachers [37]. Whereas the importance of PA to the family was a facilitator [33].

Community: Barriers at the community level included those within the constructed environment such as a lack of sporting facilities [34, 37], distance between sporting venues and residential areas [34], lack of transport [34, 37], lack of equipment [33, 40], lack of appropriate training of coaches [40] and lack of organised activities particularly for older children [33] and family oriented sports programs [36]. Safety concerns such as safety around play areas [34], fear of dogs [33], poor community cohesion [34], and experiencing racism in the community [34] were barriers. Negative community member role-modelling was also a barrier [34, 38]. Poor attendance of coaching programs and a perception that academic skills were required to participate in them acted as a barrier to providing programs [37]. Physical environment barriers included the hot climate [33] as well as seasonal restrictions such as only being able to access certain remote communities in the dry season, whereas, in a regional area, access to natural environments such as beaches and bush tracks were seen as facilitators [37, 39]. Communities not wanting to be involved in some programs was a barrier for organisations providing the programs [37]. The cost of fly-in-fly-out programs was another barrier [37].

As noted above, providing sports such as volleyball that accommodated female preferences was a facilitator [36]. Dance, including traditional dancing was also identified as a facilitator especially for girls [38]. Having well-known sports people—both Aboriginal and Torres Strait Islander and non-Indigenous—as role models was a facilitator [38]. Having organisations run the programs with the participants just ‘turning up’ was seen as a facilitator, as was having consistent mentoring of local staff to maintain motivation [37]. Community competition was both a barrier, in regard to feelings of shame associated with losing and violence in matches, and a facilitator, relating to the reward and motivation from wining [38].

The barriers for remote communities were lack of activities/programs [33], climate too hot, seasonal restrictions [37] and fear of dogs [33]. The facilitators for remote communities were having organisations provide the sporting service in partnerships with communities [37].

Policy/Enabling Environment: Continuity of program provision was also a barrier, such as one-off events like “come and try em” days or programs that were not ongoing [36]. In contrast, depending on external organisations coming into communities to run programs was seen as a barrier [34, 36, 37]. A facilitator to overcome this was establishing a partnership with the community before a program commenced [37]. Having a long-term Aboriginal community governed collaboration was a facilitator [34].

For remote areas, a barrier was lack of continuity of provision of sporting programs [36]. A facilitator was using a memorandum of understanding to make the partnership with community explicit [37].

## Discussion

This review aimed to synthesise the existing literature regarding barriers and facilitators to PA and sports participation for Aboriginal and Torres Strait Islander children and adolescents. There is a limited amount of Australia-wide research with five of the nine studies spanning only across two states in Australia. This study was focused on a comprehensive identification of barriers and facilitators at each level of the social-ecological model. From this process, gender and geographic location emerged as areas in which individual engagement with PA and sport was highly influenced by environmental, community, and policy factors. The majority of barriers were at the individual and community levels, while facilitators were highest at the individual level with interpersonal and community facilitators equally represented.

At the individual level, the most commonly reported barriers related to shyness/ embarrassment, self-perception of not being good enough as well as lack of motivation and time. Barriers to participation in PA identified in relation to girls and young women show how the complex interactions of interpersonal and cultural factors, associated with gender can be strongly determining the personal behaviour of individuals. For example, three studies reported young Aboriginal and Torres Strait Islander women feeling shy or embarrassed about wearing sports clothing, particularly bathers, and identifying it as a barrier to participation in PA [33, 36, 39]. Macdonald et al. explained that young women’s self-consciousness about wearing ‘minimal’ clothing related to them feeling they were ‘the object of the public gaze’. These authors suggest that this experience of objectification is a cross-cultural, gender-based experience wherein, ‘an ambivalent relationship with exercise and the body is not limited to Indigenous women with women world-wide feeling more alienated than men from dominant forms of physical activity and their bodies … ’ [36]. Evans et al. note that the lower levels of PA recorded for Aboriginal and Torres Strait Island girls at a national level is ‘consistent with world literature’ which finds boys participating in PA at higher rates [25]. However, it is important to recognise that two women in McDonald et al.’s study, challenged notions associated with ‘shame’ held more generally in their community: For example, Diana remembers not caring or feeling ‘shame’ about running in public when she was young, and Carina also considered ‘shame’ or feeling embarrassed about doing PA in public to be ‘old fashioned’. Carina’s ideas may indicate a process of intergenerational change in relation to the attitude and behaviours of girls and women in her community toward sport and PA. This highlights the need to consider both the broad, cross-cultural aspects of gender as well as the specific contexts of individuals when examining the barriers to participation of Aboriginal and Torres Strait Islander girls and women. Research has reiterated the importance of hearing the voices of Aboriginal and Torres Strait Islander children and young people when developing and evaluating sports and PA programs designed for them [21, 39]. This applies too to finding out from young women where and what kinds of PA they are most comfortable doing, as well as promoting and redefining traditionally male sports as suitable for females to encourage greater participation of Aboriginal and Torres Strait Islander girls, particularly in remote areas.

At the interpersonal level having family and friends who were active was one of the most commonly reported facilitators; and if they were inactive this was a barrier. This highlights the importance of participation in sport/PA for Aboriginal and Torres Strait Islander adults, in that they act as role models for children. Thus, while this paper has focused specifically on barriers and facilitators in children and adolescent, adult level barriers/ facilitators are also important given their flow on effects to young people. Providing family sporting opportunities and adult sport and PA options where parents and other family members can role model being physically active and participating, as well as leveraging transportation, may overcome these barriers. Fewer programs for older children was also noted as a barrier. Given that Aboriginal and Torres Strait Islander children engage in more PA than non-Indigenous children with this trend reversing into adulthood [13], adolescence is likely a critical time where programs are needed.

The term ‘shame’ was used by participants in a number of studies in reference to the performative aspect of sport and PA where one’s body, physical skills and status as winner or loser can be observed publicly. For some young people their perceived lack of ability or the experience of losing in a sporting competition were deterrents to participation in and enjoyment of PA and sport. For example, in a study involving urban Aboriginal and Torres Strait Islander youth, a young man expressed feelings of “shame” when he did not win. Another boy felt, “shy when boys play cos I think I’m unfit” [38]. However, in the same study the author reports ‘(s) everal young people identified dancing as an avenue for pride'—“I’m not shamed when I’m Aboriginal dancing (Julie)”. Examining these findings according to a socio-ecological model suggests the individual feelings and attitudes of children and young people towards PA and sport are significantly influenced by interpersonal and communal factors relating to their sense of how they are perceived by others. This is indicated by the strong awareness reported by some of the young people in the studies concerning their bodily appearance, physical skills, and competitive success. At the same time, it is important to note that some participants described their enjoyment of competitive sport which included being part of a team. The prospect of winning was also a motivation. Nelson comments that the young Aboriginal and Torres Strait Islander youth involved in competitive sport and dance performance in her study, ‘took opportunities to perform their Indigenous ‘selves’ as strong and powerful’ [38]. Abbott et al. have proposed deterrents to sport and PA associated with perceived lack of physical skills may be mitigated by ensuring Aboriginal and Torres Strait Islander young people have, ‘opportunities across a range of competitive and non-competitive sport and other physical activities that suit the preferences of all children’ [33]. Trained coaches and mentors could also play an important role in assisting young people to deal with self-esteem issues and emotional regulation that can arise in the process of developing physical skills and playing competitive sport with others.

Around two-thirds of the Aboriginal and Torres strait Islander population live in regional and remote areas, a rate twice that of the general Australian population [25]. This includes ‘21.4% of Aboriginal and Torres Strait Islanders who live in remote or very remote areas compared to only 1.7% of other Australians’ [41]. For the five studies conducted in regional and remote areas, barriers to participation in PA were particularly evident at community and environmental levels. These included lack of access to sporting facilities, organised PA and sport programs and transport to and from venues. Living in a ‘hot and sweaty’ climate and restrictions to travel in the wet season were also deterrents to participation in PA for young people from some communities in the Torres Strait Islands and the Cape York Peninsula. However, in the regional city of Geraldton (WA) the opportunity to engage in PA in natural environments such as parks, beaches and bush tracks was an important facilitator for young Aboriginal and Torres Strait Islanders [39]. Evans et al. also reported the ‘striking finding’ that in remote communities higher levels in PA were associated with children with a lower socio economic status. The authors attribute this to the dependence in low socio-economic status (SES) communities on non-motorised transport and rudimentary infrastructure which means ‘financial differentials which characterise sports participation in urban areas may not come into play’ [25].

The most common policy level barrier was “welfarism” where communities were dis-empowered by external organisations coordinating programs. One study provided a remedy for this by creating a memorandum of understanding to ensure programs were conducted in a partnership with the community. The continuity of programs was also a barrier, and this was also reflected at other social-ecological levels through high staff turnover.

Taken together, programs which offer sporting participation options for all family members (children, adolescents and adults), which appeal to males and females, are accessible through existing transport and related infrastructure, and are committed to communities through formal partnerships are needed. There are unique barriers in remote areas including, lack of programs in the local language, the transient nature of teachers, climate factors and a lack of (ongoing) sporting programs which will need targeted approaches to overcome. Overall, the health benefits of sport as well as its ability to provide future opportunities for young people are also a key facilitator. Public health campaigns broadening these messages to cover both physical and mental health towards Aboriginal and Torres Strait Islander People could increase participation in sport and thus derive improved health outcomes.

### Limitations and future research

The experiences of ‘shame’ or embarrassment reported by some young Aboriginal and Torres Strait Islanders in relation to feeling uncomfortable about their bodies or inadequate about their physical abilities were reported in studies with relatively small numbers of participants. Of these, one study was solely focused on exploring barriers. Facilitators were under-researched relative to barriers. Only two studies included an Australian population representative group. Most Australian states were not included in any of the remaining studies, nor was the Northern Territory or the Australian Capital Territory. Three studies explored remote areas, one an urban area in Queensland, and two were conducted in regional areas of Western Australia and New South Wales respectively. Thus, urban and regional perspectives were somewhat lacking, and only remote Queensland locations were explored. Each area, even within the same area classification, may have unique barriers and facilitators. It is also important to consider that sport is not a ‘panacea’ that can rectify health, education and social disadvantage in Aboriginal and Torres Strait Islander communities [19]. Stereotypes of Aboriginal and Torres Strait Islander People being intrinsically good at sport can themselves act as a barrier to young Aboriginal and Torres Strait Islanders engaging in sport and PA [19, 38]. This was perhaps reflected in the individual-level barrier wherein some young people perceived themselves as lacking sporting ability. Careful messaging and the provision of services to offer young Aboriginal and Torres Strait Islanders a breadth of cultural, educational and recreational opportunities are needed so these stereotypes are not perpetuated.

Further research needs to build on the evidence for the link between sport/PA participation and improved outcomes across a range of individual and community health indicators in Aboriginal and Torres Strait Islander People. This research will need to cover a range of educational, cultural and geographic settings as well as sports/PA programs operated through public and private service providers.

Given the switch from Aboriginal and Torres Strait Islander children engaging in more PA than non-Indigenous children, to less PA participation than their non-Indigenous peers in adolescence, further exploring the barriers and facilitators in this age group is of particular importance [13]. The included studies did not provide separate information on barriers and facilitators for children versus adolescents but this will be important for future studies to understand. Future research exploring how to recruit and retain adolescents into adult PA and sport participation is needed to counteract the trend for Aboriginal and Torres Strait Islander adults to engage in less activity than non-Indigenous adults. These young people may then become role models and positively influence the younger generation to take part in sport and PA. Exploring the intersection with disability as another potential barrier may also be significant given the higher levels of physical and learning disability in Aboriginal and Torres Strait Islander children relative to their non-Indigenous peers [38].

The cultural and geographic diversity of Aboriginal and Torres Strait Islander individuals and communities points to the value of further research such as that recently undertaken by Dahlberg et al. which foregrounds the perceptions of Aboriginal and Torres Strait Islander People themselves in order to understand their attitudes and beliefs regarding PA and sport. The barriers and facilitators of sport and PA identified through this social-ecological review indicate the importance of understanding the interactions between individual, community and cultural factors particularly in relation to the gender and geographic location of young Aboriginal and Torres Strait Islander Peoples [21].

## Conclusions

Participation in sport and PA could be leveraged to minimise the increased risk of poor socio-emotional wellbeing and physical health outcomes for Aboriginal and Torres Strait Islander children and adolescents. This review of the barriers and facilitators for Aboriginal and Torres Strait Islander children and adolescents showed that research in this area is limited with some states in Australia not represented and few participants involved across studies. Hence, barriers and facilitators are not fully known in many communities. The Australian Government’s Closing the Gap initiatives have included a framework for sports service delivery which highlight the need to ‘collect comprehensive data on Indigenous participation’ and to ‘prioritise strategies to increase opportunities for Indigenous female participation’ [26]. Studies are needed to understand whether this initiative from 2013 has influenced practice. Strategies and programs for improving participation in sport and PA for Aboriginal and Torres Strait Islander children and adolescents need to be evidence-based and take into account the unique contexts of young people in their communities.

## Data Availability

All data is included within the manuscript file.

## Abbreviations

AFL: Australian Football League
MMAT: Mixed methods appraisal tool
NRL: National Rugby League
NSW: New South Wales (Australia)
PA: Physical activity
SES: Socio-economic status
WA: Western Australia
